# Neurorehabilitation for Multiple Sclerosis Patients with Emotional Dysfunctions

**DOI:** 10.3389/fneur.2015.00272

**Published:** 2016-01-25

**Authors:** Yuwen Hung, Pavel Yarmak

**Affiliations:** ^1^Martinos Imaging Center, McGovern Institute for Brain Research, Harvard-Massachusetts Institute of Technology, Cambridge, MA, USA; ^2^Neurosurgery, Neuroscience Research Center, St. Michael’s Hospital, Toronto, ON, Canada; ^3^Psychology and Neuroscience, University of Toronto, Toronto, ON, Canada

**Keywords:** multiple sclerosis, emotional disorders, neuropsychiatry, psychotherapy, neurocognitive rehabilitation

## Abstract

Depression frequently develops in multiple sclerosis (MS) patients, exacerbating the manifestations of the disease and making its management challenging. To date, no consensus has been reached regarding effective treatments for these sufferers due to limited understanding regarding the underlying mechanisms responsible for emotional disorders that are highly comorbid with this disease. There is an urgent need to rethink current treatment options for these patients. This article aims to optimize the treatment outcomes and improve the quality of life for MS patients. Based on an in-depth and critical review of the current literature, we provide a neurorehabilitative framework that explains possible regulatory mechanisms underlying the emotional symptoms highly developed in MS. This article offers practical knowledge and therapeutic strategies to optimize the treatment options in the current care system for MS, as well as for other disabling diseases.

Approximately half of all patients with multiple sclerosis (MS) experience clinically significant depression at least once in their lifetimes and even more exhibit emotional symptoms. The depressive disorders observed in MS exacerbate the manifestations of the disease and make its management challenging (1). Yet, the majority of MS patients with depressive symptoms receive neither antidepressant medication nor psychotherapy. The side effect profile of conventional antidepressant treatments can complicate MS management, compromising treatment efficacy and compliance (2). No consensus to date has been reached regarding effective treatment options for these sufferers, due to limited understanding regarding the underlying mechanisms that may be responsible for the emotional disorders that are highly comorbid with MS. There is therefore an urgent need for clinicians and neuroscientists to rethink current treatment options for these patients and improve the quality of their care. Here, we provide a neurorehabilitative framework that explains possible regulatory mechanisms underlying the emotional symptoms frequently developed in MS and suggest practical strategies to optimize its treatment outcomes.

Patients with MS, a heterogeneous central nervous system disease causing focal brain lesions and diffuse demyelination, suffer from pronounced physical and cognitive disabilities (3). Though retaining their intellectual abilities, MS patients exhibit various cognitive deficits involving both verbal and non-verbal memory, attention and speed of processing, as well as executive functioning. The progression of MS introduces a chronic stress in patients, associated with impaired neurocognitive functions and diminished brain resources (cognitive reserve), which involve pathology of not only the cortex but also deep brain structures, including the limbic system. In particular, impaired brain connectivity in MS patients has been observed between the prefrontal lobe and the amygdale (4) – brain circuits important for the regulation of emotions (Figures 1A,B) (5). Deficits in executive functions also contribute to problems of impulsivity and lack of emotional control. Abnormal emotional processing takes place in the dysregulated brain with limited neurocognitive resources, and the MS patients experience negative attentional bias and use maladaptive cognitive appraisal toward daily life events. These cognitive deficiencies create a susceptibility to the development of emotional symptoms and disorders such as depression (Figures 1B,C). Neuroimaging evidence suggests that the depressive symptoms in MS are related to the total extent of brain lesions and the degree of impaired cortical-subcortical connections (6).

**Figure 1:**
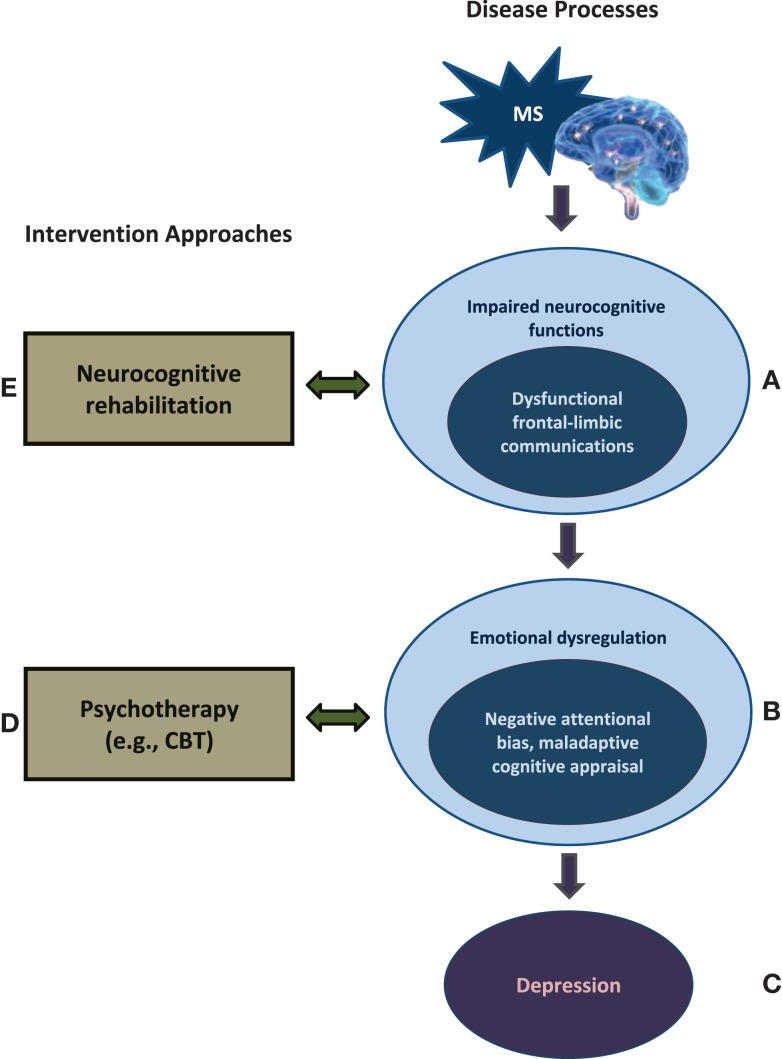
**A neurorehabilitative approach for optimizing the MS treatment**. **(A,B)** MS introduces a chronic stress, associated with impaired neurocognitive functions and reduced brain resources, which involves cortical–subcortical communications – particularly the prefrontal-amygdala connections, areas important for emotional regulation. **(B,C)** MS patients experience negative attentional bias and maladaptive cognitive appraisal toward daily life events because of abnormal processing and insufficient cognitive resources in the emotionally dysregulated brain, which create a susceptibility to the development of emotional dysfunctions. Given the complex relationship between the neurocognitive dysfunctions and depressive susceptibility in MS, it is advisable to combine psychotherapy **(D)** with neurocognitive rehabilitation **(E)** in future MS treatment options.

This framework lays an important foundation to explain how neuropsychotherapy can be an alternative to antidepressants for MS patients with active depressive symptom. Specifically, based on the complex interplay between the neurocognitive dysfunctions and depressive susceptibility in MS, it is advisable to consider combining psychotherapy with neurocognitive rehabilitation in current MS treatment options to optimize treatment outcomes. It has been found that emotional distress in the MS patients is associated with poor coping and acceptance rather than disease duration or severity (7). Stress management and better coping strategies have been shown to be related to increased psychological well-being in people with MS (8). Emotional reappraisal strategies, regardless of the severity of the disease, can further improve the quality of life with MS (9). Cognitive-behavioral therapy (CBT) has recently been found to be an effective means of treating depressed MS patients (10, 11). This psychotherapy enables the top-down, prefrontal-related regulatory processes to mitigate negative emotional reactions (12), with an efficacy similar to antidepressant treatments. CBT corrects the MS patients’ negative attention and perception toward daily challenges as well as adjusting their maladaptive ways of thinking – negative cognitive appraisal – which eventually produce a depressed mood (13). This intervention can help patients with MS learn to cope with existing and continuing cognitive impairments by adopting a more realistic cognitive frame to lower their stress levels when negative situations cannot be avoided (Figure 1D).

Lastly, neurocognitive rehabilitation treatment can be effective in ameliorating patients’ cognitive deficiencies (14), both objectively and subjectively, while relieving the depressive symptoms. The rehabilitative intervention aims to retrain the impaired cognitive functions and allow the brain to maximize cognitive capacity (reserve) or to utilize a compensatory network (neural plasticity). Depending on the cognitive symptoms, the rehabilitative exercises target functions including attention (e.g., focusing), memory, and working memory, as well as executive functions and learning strategies (e.g., goal attainment). Future cognitive training may benefit from considering higher-level, prefrontal-related executive processing to strengthen the cognitive controls and facilitate regulative processes for affective functioning (e.g., impulse control including inhibition and attention training involving attention shifting and disengaging) (Figure 1E). The cognitive retraining for MS patients may also facilitate the effects of CBT through positive feedback that increases the patients’ self-efficacy (self autonomy). Currently, cognitive rehabilitative exercises have not been well established for patients with MS, relative to other neurological diseases (e.g., stroke), possibly due to the highly varied neuropsychological profiles among individuals with MS.

Given the emotionally afflicting nature of MS and the high risk of patients developing affective dysfunctions, it is also important to consider early, preventative intervention, before a definite diagnosis of depression is made. Such intervention can include adding screening tests for initial signs of depression and identifying at-risk MS patients (e.g., those with emotional dysregulation symptoms). These directions would not only facilitate treatment outcomes for the MS patients, but would also potentially lower the substantial incidence of depression in MS. For future research and treatment planning, a multimodal approach may be the key to optimizing treatment outcomes for many people suffering from MS and related diseases (15).

## Author Contributions

PY contributed to the conception and drafting of the work for important intellectual content. YH contributed to the design, conception, and supervision of the work, and revising the manuscript for manuscript submission.

## Conflict of Interest Statement

The authors declare that the research was conducted in the absence of any commercial or financial relationships that could be construed as a potential conflict of interest.
